# How can mobile phones be used to improve nutrition service delivery in rural Bangladesh?

**DOI:** 10.1186/s12913-018-3351-z

**Published:** 2018-07-09

**Authors:** Nazib Uz Zaman Khan, Sabrina Rasheed, Tamanna Sharmin, A. K. Siddique, Micheal Dibley, Ashraful Alam

**Affiliations:** 1 International Centre for Diarrhoeal Disease Research, Bangladesh (icddr, b), 68 Shaheed Tajuddin Ahmed Sarani, Mohakhali, Dhaka, 1212 Bangladesh; 2 International Public Health, Sydney School of Public Health, Sydney Medical School, The University of Sydney, Sydney, Australia

**Keywords:** IYCF, Nutrition, Formative research, Mobile phone, Bangladesh

## Abstract

**Background:**

Nutrition has been integrated within the health services in Bangladesh as it is an important issue for health and development. High penetration of mobile phones in the community and favourable policy and political commitment of the Government of Bangladesh has created possibilities of using Information Communication Technology such as mobile phones for nutrition programs. In this paper the implementation of nutrition services with a specific focus on infant and young child feeding was explored and the potential for using mobile phones to improve the quality and coverage of nutrition services was assessed.

**Methods:**

A qualitative study was conducted in Mirzapur and Chakaria sub-districts, Bangladesh from February–April 2014. We conducted 24 in-depth interviews (mothers of young children), 8 focus group discussions (fathers and grandmothers); and 13 key informant interviews (community health workers or CHWs). We also observed 4 facilities and followed 2 CHWs during their work day. The data was analyzed manually using pre-existing themes.

**Results:**

In this community, mothers demonstrated gaps in knowledge about IYCF. They depended on their social network and media for IYCF information. Although CHWs were trusted in the community, mothers and their family members did not consider them a good source of nutrition information as they did not talk about nutrition. In terms of ICTs, mobile phones were the most available and used by both CHWs and mothers. CHWs showed willingness to incorporate nutrition counselling through mobile phone as this can enhance their productivity, reduce travel time and improve service quality. Mothers were willing to receive voice calls from CHWs as long as the decision makers in the households were informed.

**Conclusions:**

Our study indicated that there are gaps in IYCF related service delivery and there is a potential for using mobile phones to both strengthen the quality of service delivery as well as reaching out to the mothers in the community. It is important however, to consider the community readiness to accept the technology during the design and delivery of the intervention.

## Background

Globally, remarkable progress has been made in reducing child mortality. The mortality of children under 5 years of age has declined by 53% between 1990 (91 deaths/1000 live births) and 2015 (43 deaths/1000 live births) [1] . Bangladesh also made significant gains in child survival between 2007 and 2014 as reflected by a decline in mortality among neonates (24.32%) infants (27%) and children under 5 years of age (29.23%) [2]. Despite these gains, globally, an estimated 5.9 million children under the age of 5 years died in 2015. Nearly half of these deaths were attributable to malnutrition [3]. Over two-thirds of these deaths from malnutrition (~ 2 million) were associated with inappropriate infant and young child feeding practices (IYCF) that occurred during the first year of life [3]. In South Asia, home to 26% of the world’s under five child population, 38% are stunted, indicative of wide spread malnutrition [4]. In Bangladesh 36% of children < 5 years of age or 5.4 million children are stunted [2].

With the recognition of the importance of nutrition for national development, nutrition has been integrated within the health services in Bangladesh [5]. Nutrition has been mainstreamed within existing health and family planning service delivery platforms although some gaps in the policy environment remain for IYCF [6]. Furthermore, these platforms face challenges regarding coverage, access, resource and quality. Hence implementation of IYCF service delivery through these platforms remain inadequate [7] . In previous studies conducted in Bangladesh and elsewhere, face-to-face counseling by peer counselors [8, 9] and frontline health workers improved IYCF practices in the community [10, 11]. However, for sustained and national impact, it is important that the public health system is strengthened to provide good quality nutrition information and ensure adequate coverage of services.

Information Communication and Technology (ICTs) tools such as mobile phones and computers have been touted to circumvent health system bottlenecks such as access, coverage and resource gaps [12]. Mobile phones have been successfully used to inform, support and empower community health workers [13] in Bangladesh and other developing countries. Among community members mobile phones have been used successfully to deliver health messages, provide emergency medical assistance, decision support and referral to providers and services [13–16]. These factors along with increasing numbers of ICT users [17] have helped in rapid proliferation of mHealth and eHealth interventions globally and in Bangladesh [18]. In Bangladesh with the backdrop of enabling policy mandate [19], laptops and tablets were provided to the frontline public health workers for collecting data on health indicators for Health Management Information System [20]. Therefore, there is an opportunity to use ICTs to energize nutrition service delivery in the public sector [21].

In this paper, the potential for using ICT tools such as mobile phones to improve the quality and coverage of community-based nutrition services was explored.

## Methods

### Public sector health structure and frontline health and nutrition workforce

The Ministry of Health and Family Welfare has an extensive health infrastructure. In rural areas frontline health and nutrition services are provided through two directorates - Director General of Health Services (DGHS) and Director General of Family Planning services (DGFP). To deliver primary care, DGHS has established Community Clinics (CC) that provides basic healthcare to people in the community. Full-time community healthcare providers (CHCP) have at least 12th grade education and 6 months of basic training on health care. They provide services in the CCs for 6 days a week. The domiciliary staff of DGHS (Health Assistant or HA) and DGFP (Family Welfare Assistants or FWAs) have at least 10th grade education and on the job training. They provide services at the CC 3 days a week. HA is mostly responsible for immunization while FWA provides family planning services. As an outreach activity HA is responsible for satellite clinics for immunization where FWA assists. FWA conducts house visits to provide family planning services. DGFP has also established union health and family welfare centers (UHFWCs) where paramedics and Family Welfare Visitors (FWVs), with at least 10th grade education and 18 months of midwifery training provide antenatal and postnatal care, child health care, contraceptives and treatment for general patients. Health, nutrition education and counseling is also part of their services [2].

### Study locations and population

Data was collected from two sub-districts Mirzapur and Chakaria. Mirzapur is a semi urban sub-district nearly 50 km away from the capital city Dhaka. Chakaria is a remote rural sub-district, situated in the South Eastern part of Bangladesh. Mirzapur has a population of about 3.7 million with population density of1057/ sq. km whereas, the population of Chakaria is 2.3 million with a population density of 909/ sq.km. Nearly 15% area of Mirzapur is urban compared to 21% of Chakaria. The average literacy rate in Mirzapur is 47% compared to 39% in Chakaria. However, agriculture is the major occupation in both areas [22, 23].

### Data collection

Two unions with no shortage of frontline staff were randomly selected from each field site. To select the respondents, a list of pregnant and lactating women was collected from the CCs of the selected unions. Respondents with characteristics to capture maximum variation of experience were selected from the list. Recruitment was continued until data redundancy was reached. The respondents were mostly mothers of small children, pregnant women, older female family members (mother-in-laws) and fathers. All the available frontline health workers were interviewed.

### Data collection

The qualitative methods used for data collection were in-depth interviews (IDI) and focus group discussions (FGD) for community members, and key informant interviews (KII), observations (community clinics, home visits and immunization sessions) were conducted with CHWs (Table 1).

**Table 1:** Methods used and sample size

Methods	Types of respondents	Number
In-depth interview (IDI)	Teen age pregnant women	6
	Women with 7–9 months pregnancy	6
	Women with 0–12 month old child	6
	Women with 12–24 month old child	6
Key informant interview (KII)	Community health care provider (CHCP)	3
	Family welfare visitor (FWV)	4
	Family welfare assistant (FWA)	4
	Health Assistant (HA)	2
Focus group discussion (FGD)	Grandmothers	4
	Fathers	4
Following CHW during workday	Health Assistant (HA)	2
	Family welfare assistants (FWA)	2
Observation	Family welfare centre (FWC) and Community clinic (CC)	4

Mothers’ knowledge and practice regarding infant and young child feeding, experience of interacting with health care providers and use of mobile phones were explored through IDIs. FGDs were conducted to understand the community norms from the perspectives of mothers-in-law and husbands regarding their role and involvement around IYCF, use of mobile phones as well as opinions about mothers receiving IYCF information and counseling over phone. With CHWs, we used IDIs to explore their knowledge and training related to IYCF and their ICT use for official and personal purposes. For both mothers and CHWs, we explored the potential of a program where IYCF messages and counseling can be delivered through mobile phones. In addition we observed the community clinics to understand the context in which IYCF counseling was currently implemented. We also followed the CHWs to understand the norms related to nutrition counseling during field visits.

Four researchers (2 males and 2 females) with training in anthropology and extensive experience in qualitative data collection were employed. They were trained and supervised by trained anthropologists (TS, NK, AA) and a nutritionist (SR). The interviews took place at the respondents’ home at the time of their preference. Each interview took 45 min to an hour to complete. Field notes were also taken to record the observations around the interviews.

Data was collected from February to April 2014.

### Data analysis

All the interviews were audio-recorded and transcribed in Bengali for analysis by the researchers themselves. During data collection, field diaries were maintained and day-to-day field experience was recorded.

**Fig. 1:**
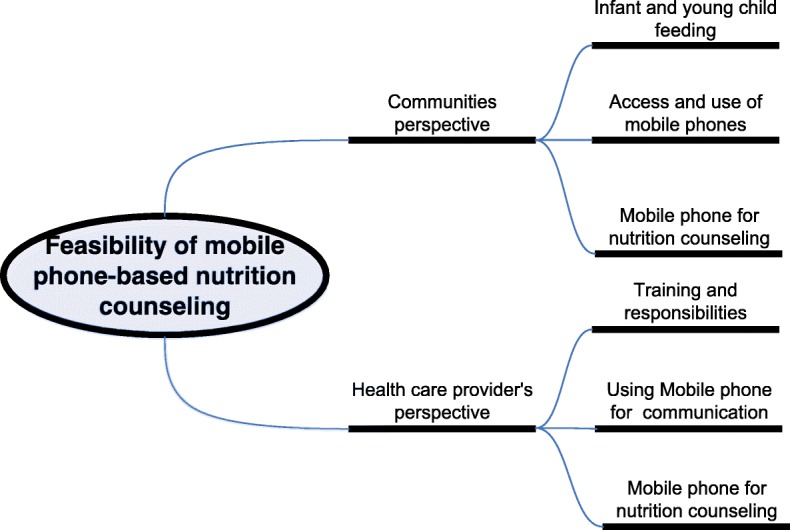
Code tree

A thematic approach was used for analyzing the data. During analysis, salient themes and sub-themes were identified. The common patterns that emerged through all interviews were identified and the atypical patterns were noted to accommodate the diversity of meanings and to generate new insights and typologies for further exploration. Themes were triangulate using data from IDIs, FGDs and observations. The members of the research team discussed the themes amongst themselves to come to consensus about the different codes. The codes and sub-codes are displayed in the code tree (Fig. 1). Regular feedback sessions with the research team were conducted as part of the peer de-briefing to understand the issues and consolidate the findings. The quotes were translated into English verbatim during paper writing.

## Results

The participating mothers were between 16 and 36 years of age and had 6–10 years of schooling. Most mothers were multipara and very few were employed outside the home (Table 2). The CHWs, mostly females, were between 24 and 56 years of age; had more than 10 years of education and served in government between 2 and 27 years (Table 2). The CHCPs were the youngest group of workers and were mostly unmarried.

**Table 2:** Characteristics of the respondents

	Number	
Characteristics	Mother % (n) *n* = 24	CHW% (n) *n* = 13
Age (y) range	16–36	24–56
Parity		
Primipara	25 (6)	No data
Multipara	75 (18)	No data
Sex		
Male	0 (0)	15 (2)
Female	100 (24)	85 (11)
Schooling (y)		
0–5	38 (9)	0 (0)
6–10	62 (15)	31 (4)
10+	0	69 (9)
Occupation		
Housewife	88 (21)	0 (0)
Employed	22 (3)	100 (13)
Duration of Service (y) range	No data	2–27

### Community’s perspective

#### Infant and young child feeding

Mothers knew that colostrum should be fed to infants after birth but did not know the ideal time of breastfeeding initiation. They did not have a clear idea about the problems associated with feeding prelacteals and usually fed honey, mustard oils and sugar water to their infants. All the pregnant and lactating women and their mothers preferred breast milk over formula. As one mother clearly stated:

“*Formula milk contains less nutrition compared to breast milk”* (Mother of 0–12 month old child)

However, many mothers started bottle feeding with Lactogen-1 (a local brand of formula) before the child was 6 months old due to perception of breast milk inadequacy. When the mothers felt that they were not producing adequate milk they consulted people within their network such as peers, neighbors, experienced older women in the family. They also consulted local pharmacies or village doctors rather than CHWs. The people mothers reached out advised the use of complementary foods or other strategies to improve breast milk production. Mothers then picked the most suitable option for themselves. As a mother explained:

“*She was crying a lot ... not getting enough (milk). My aunt suggested Cerelac (tinned complementary food) or khichuri (home-made complementary food). She also suggested that I eat more. My child is small and has trouble swallowing khichuri so I bought Cerelac*.”

Some mothers who delivered their baby in the health facility or took infants to a health facility during illness were provided information related to infant feeding by the doctors and nurses.

“*He (the baby) had both breast milk and formula. He wasn’t getting enough (breast milk). The doctor said that Lactogen is good, so I bought it. No the doctor did not prescribe... people said this was a good milk.*” (Mother of 12–24 month old)

Mothers consulted their peers or trusted members of their social network for advice on complementary feeding. TV and radio advertisements were also mentioned as a source of information about complementary feeding. As a teen age pregnant woman said very eloquently

“*I heard breast milk has to be fed for 6 months. Afterwards I mean, have to give extra food… egg, fish, liver this type of food has to be fed. They are good for the baby’s brain and health. I have heard this on TV... I also heard about these things from my sister-in-law and aunt*.”

Mothers did not consider CHWs as an important source of IYCF information as they were seen to *“*only provide medicine*”.*

#### Access and use of mobile phone

Only 7 out of 24 mothers owned personal mobile phones and therefore, access to mobile phone was an issue. Mothers who did not own mobile phones spoke about using their neighbor’s phones during emergencies. Sometimes mobile phones were owned by male family members who carried them to work. The women could use it when the males came home in the evening after obtaining permission. As one lactating mother stated:

“*When my husband comes (at night) I can use the mobile phone if it is an emergency*.”

Cost was an important consideration for mothers when using mobile phones. Mostly mothers used mobile phones for missed calls and receiving calls from others as this did not cost money. Only one mother whose husband lived abroad mentioned calling people with her phone. Mothers depended on their husbands for top-up money which also limited their use of mobile phones. As one teenage female respondent said,

“*I call my mother; I give missed (blank) calls if I don’t have money. My mother calls back. Women sometimes don’t have money, men do. My husband calls every day. If I provide the number (own number) and money, he can buy balance (in the phone) for me. I don’t spend much.... maybe 50 taka a month*.”

Among those who had access to mobile phones and used them, the usage was limited to voice calls. Few mothers mentioned that they call health workers for appointments. A few mentioned calling the responsible person in the health facilities to find out whether the facility is open or closed. But none of the respondents mentioned calling a CHW for any nutrition information. Very few (better educated respondents) mentioned that they were actually able to read SMS although they usually did not read them.

### Health worker’s perspectives

The CHWs had some basic training on nutrition and a few had training on the use of laptops. The CHWs’ responsibilities and workloads were explored from their perspective to understand whether they viewed nutrition to be a part of their responsibilities. Their current use of ICTs was also explored.

#### Training and responsibilities of frontline health workers

The CHWs interviewed were based at the Community Clinic (CC) and Family Welfare Centre (FWC). Among the CHWs, HAs and FWAs went out to the community and conducted household visits while Community Health Care Providers (CHCPs) and Family Welfare Visitors (FWV) were based at the static facilities. Nutrition was a part of the 3 month basic training of all CHWs although the HA did not specifically mention it (Table 3). It is important to note that only CHCPs mentioned nutrition as a part of their daily activities. In terms of ICTs, CHCPs reported being trained on the use of laptops as they were responsible for entering data through official laptops (Table 3).

**Table 3:** Community health workers, their training and responsibilities (self report)

CHW type	Types of training		Responsibilities/activities
	Nutrition	ICT	
CHCP (Female) 10+ years of schooling	Nutrition training was a part of the 3 month foundation training. Received specific training on IYCF	Received 4–5 day training on how to use laptops for data entry	- Registration of newly married couple, pregnant women, birth & death and records of expected date of delivery - Treatment of minor primary health problems - Nutrition counseling for pregnant women and lactating women
FWV (Female) 10+ years of schooling	Nutrition was part of the 6–18 month midwifery training	No training received	- Antenatal and post natal care - Nutrition message for pregnant women - Treatment of minor primary health problems - Delivery assistance
FWA (Female) 10+ years of schooling	Nutrition was a part of 6 month foundation training	No training received	- Providing information related to contraceptives - Providing information on immunization day or camp through home visit
HA (Male) 10+ years of schooling	Basic training for 21 days on primary health care and immunization	No training received	- Providing information regarding vaccination camp - Providing vitamin A supplementation after delivery to mothers and children of under 5 years of age - Providing tetanus toxoid injection for 15–49 years old females

During observation of static health facilities some posters on nutrition were noted on the walls of the CCs. Some teaching materials were available in the cupboard in one CC that the CHW was able to show. However, use of the teaching materials during encounter between mothers and CHWs was not observed. During an interview one CHW mentioned that she provides IYCF information to mothers when they ask for it. This finding was supported by observation that mothers of small children, when they came for a sick child visit, did not specifically ask for IYCF information. Only once a CHW was seen to talk to a pregnant woman about feeding colostrum during an antenatal visit (Table 4). The opportunity of IYCF counselling was further limited by a lot of women arriving at a time for service both in the static facilities and the vaccination centres. In terms of the use of official laptops and tablets, although CHWs reported having them, the presence of the machines or their use in the facilities was not observed. The CHCPs explained that the absence of electricity in the facility and lack of secure storage space for laptops were barriers for them to bring the laptops to work.

**Table 4:** Results from observation of CCs and FWCs

Topic	Observation
Availability and use of ICTs	- Personal mobile phones were seen - Only one mother called FWA to find out her availability
BCC and job aid on nutrition	- Two posters on general nutrition advice during pregnancy and breast feeding week was observed in a FWC and a CC - In one CC, CHCP showed a calendar of IYCF information which was locked in a steel cupboard
Nutrition counselling	- In the FWC pregnant mothers were told about colostrums feeding - No advice was given regarding nutrition during home visits
Workload *Static facility*	- Work hours were between 10 am to 1-2 pm - CHCP was mostly present and sometimes other health workers were present. - A range of 2–20 patients, mostly women visited with complaints about common illnesses (cough, cold, fever, diarrhea, stomach ache, gastric problems etc.) - Registration happened manually through register books. When 3–4 people came at a time, registration did not occur for everyone
*Household visit*	- Talked about family planning (FWA) during home visits - Informed mothers about vaccination dates and venues (HA) during home visit
*Satellite clinic* (HA)	- Vaccinated children - Filled out the children’s vaccination card - Filled the registration book for vaccines

#### Using mobile phone for communication

All the CHWs had their own mobile phones. They used personal phones to call their supervisors for leave of absence, refer patients and seek suggestions. Prior to an immunization or health camp, CHWs used mobile phones to inform community leaders about the camps so that they could inform mothers in their communities. Only two young and educated CHWs reported reading SMS (short message service) which consist of health information from the government. A few of them complained about not receiving payment for phone bills from the office despite using phones for official purposes.

From the CHWs it was found out that community members called them over the phone to avail their services although we did not observe this. According to the CHWs women who live far away called to inquire whether the service provider would be available so that they can come for a visit. In terms of health information, FWAs told us that women call them for information on contraceptives. Only one CHW mentioned that sometimes they received calls from the mothers seeking advice about lack of appetite among children. The CHWs did not send text messages to mothers as they did not think this is a good mode of message delivery. As one family planning worker said

“*Mothers are busy with work and don’t have interest. They prefer direct calls. Many (community mothers) are not able to read the text and most of the time there is nobody to read for them*” (FWV).

#### Mode of reaching the community

When we asked for CHWs opinion about providing nutrition counselling for mothers in their communities using mobile phones, they were enthusiastic. The CHWs talked about working from 9 am to 2 pm in the static facilities which did not allow them much opportunity for counselling or message delivery. It was hard for the CHWs to provide counselling to mothers when they visited the facilities as they mostly came for treatment or specific medications. As one CHW from Chakaria said,

*“Women need to walk a long way .... so they don’t come. When they badly need medicine, they come... then they ask for everything”*

### Mobile phone for nutrition counselling

#### Person they can trust

In this section we have compiled opinions and suggestions that the community members and CHWs provided about using mobile phone for providing IYCF counselling. Mothers talked about getting nutrition information from CHWs in the past and felt the need for a good source of such information. Although at present the CHWs are not seen as a source of nutrition information and they don’t perform home visits very often any more, the mothers and other community members told us that they were trusted community members and would be acceptable sources of health and nutrition information. When imparting nutrition information it is very important that the source of information is trusted by the recipient.

*“I think it’s important that the call is from the community clinic. We know about this (facility). For example, if someone from Kumidini (renowned local hospital) calls, people will trust. It will be good.”* (Female FGD)

#### Mode of communication

Mothers preferred direct calls from CHWs as they are well known in the community. The CHWs also preferred voice calls via mobile phone to contact women rather than SMS as they perceived that women will not be able to read them as they were mostly illiterate. Only one CHW said that it is better to send text messages because the message could be saved in the inbox and when mothers had time or inclination they could read it or take the phone to someone who would read it for her.

Some mothers spoke about specifics such as that the phone number from which the calls will be made (to the mothers) should be saved under the name of the local CHWs (in the mother’s phone) and all calls should come from this number. The husbands of the women also expressed similar sentiments.

*“They (females) won’t understand if it’s not a voice call. When you call, they will understand (that someone called) and pick up. Then you can say that you are a health worker who would like to talk about health. Then they will listen.”* (Male FGD)

Mothers preferred a female voice speaking in local dialect for direct calls as it would be acceptable to their husbands and other family members. According to CHWs however, male or female voice does not make any difference as counselling will be considered medical advice.

#### Timing of the calls

Mothers chose evening or night as a good time for receiving the calls as their husbands and their phones were available at that time and mothers were also relatively free to give attention to the call. As a mother of two from Mirzapur explained

*“My husband will come and tell me... at night. He is a man.... he needs to go to many places (for work). So please call after 8pm (to get me).”*

The CHWs also suggested that the mothers be reached through the husband’s phone or when the husband was available in the house so that he could be sure of who is calling his wife.

#### Engaging the decision makers

Prior to the launching of the program mothers requested that household decision makers such as mothers in law and husbands were informed about the purpose of the project. They also need to be informed that nutrition information will be provided through voice calls to the mothers. Women felt that their husbands being the “*guardians of the family*” and paying all the bills, therefore, had the “*right to know everything”*. The CHWs also felt that it would be important to engage the main decision makers within the household for such a project to be successful. As many women do not own mobile phones, going through the husband will ensure that permission is obtained from the principal gatekeepers.

A few husbands showed interest in actively engaging with the project. They wanted to receive nutrition information and mentioned that they were quite happy to share health information with their wives although women did not talk about husbands doing this kind of sharing. As one male respondent from Mirzapur said,

*“We often don’t remember when to take the baby for Vitamin A. The campaign does not reach us... if we receive an SMS alert we can remind our family or call our wives. We can say ‘did you go for vitamin A? Go with the baby now.’”*

#### CHWs perceived benefits of using mobile phone

The CHWs perceived benefits of using mobile phones in terms of their existing work. The CHWs felt that they could provide information about vaccination camps and other services straight to the mothers if mobile phones could be integrated in to their work which would increase health service utilization in turn. The CHWs mentioned that during their scheduled home visits, they were able to only visit a few mothers but mobile phones could reduce the travel time and allow them to access more mothers with services. As one CHW said-

*“I think this is a good idea. It will save time and money ...like if it is far (the respondent’s house) you need to spend on rickshaw fare.... this money could be saved if we can call. So I think this is a good idea.”*

They also mentioned that mobile phone could allow them to call mothers after their work hours so that CHWs could provide information based on the mothers’ needs. However, if mobile phones are to be used extensively, the bills will have to be borne by the project.

Qualitative enquiry revealed that CHWs need to acquire the necessary knowledge and skills to provide adequate information and support to the mothers that help with their particular nutrition problem. For the CHWs building capacity regarding IYCF counselling and skills to deliver nutrition messages during their usual contacts with the mothers are important needs where mobile phone-based solutions could help by providing integrated training modules for the CHWs. Mobile phones can also be used to remind CHWs about appropriate nutrition messages to deliver along with their health and family planning work. From the qualitative inquiry it was quite clear mobile phones are the most available device which are already owned and used by the CHWs and mothers (Table 5). Most of the mobile phones are not smart phones and are used mostly for calling and receiving voice calls. Both community members and CHWs preferred voice calls rather than SMS as mode of message delivery. However, issues of cost for the CHWs and access for the mothers need to be addressed. To engage with the community using mobile phones, it is important to create opportunities for trust building with the household decision makers and design a responsive system based on community needs to gain acceptance.

**Table 5:** Implications for intervention

Areas of interest	Barriers	Facilitators	Opportunities for intervention
Nutrition	- Inadequate training - Nutrition counselling is not well understood by the CHWs - CHWs lack understanding of how to integrate nutrition in the existing work load. - Mothers did not see CHWs as a source of nutrition information	- CHWs have opportunities to interact with the mothers - CHWs need to report on nutrition indicators - CHWs want to provide nutrition advice	*Content* - CHWs should be adequately trained to provide nutrition counselling - There is a need for job aid that allows CHWs to integrate nutrition within existing work *Process* - Increase awareness of responsibilities regarding nutrition - Supervisors of the CHWs should be included in the process
ICT	- Inadequately trained to use ICTs - Infrastructure is not supportive for use of computers and other devices in static facilities - Not compensation provided for using mobile phones for work - Most CHWs can use mobile phone in a limited fashion (direct calls)	- CHWs already use personal mobile phones for work - Mothers call the CHWs for information - CHWs are willing to use mobile phones for nutrition - They believe use of mobile phone can save money and time	*Device* - Mobile phones are the most available device for use for CHWs *Process* - There may be training needs if CHWs need to make more than voice calls. - If mobile phones are used for program, airtime should be paid for
Community	- Many mothers do not own mobile phones - Permission from husbands needed for the CHWs to call mothers - Mothers do not use SMS	- Mothers have a need for nutrition information - Husbands are willing to share their phone with wives for health information - CHWs are trusted in the community	*Device* - Mobile phones can be used reaching mothers with nutrition counselling *Process* - Voice calls are preferred - Calls have to be made when phones are available at home - Trust building exercise has to be conducted to link the system to the community

## Discussion

Despite integration of nutrition into the health systems of Bangladesh it was found that nutrition services in the public sector need to be improved. Similar findings were reported by other researchers [24]. According to our study, frontline healthcare workers already own and use mobile phones for work and were enthusiastic about using them formally for nutrition. It was that the cultural context and power relations related to access and use of mobile phones need to be addressed to reach mothers with mobile phones. Despite high levels of mobiles phone ownership in Bangladesh [25, 26] other researchers have reported the existence of digital divide in terms of gender, education and socioeconomic status of the household [27, 28] as well. This study is the first to describe the opportunities of using ICT tools such as mobile phones in improving nutrition service delivery at community-level from both provider and consumer perspectives in Bangladesh.

Frontline health workers are a trusted source of health information and service and provide a bridge between the community and the health systems [29, 30]. Our study showed that despite being trusted, CHWs were not seen as an important source of nutrition information and support. Therefore, there is an opportunity to help the CHWs become nutrition focal points for the community. According to our data CHWs already used their own mobile phones for their work. Therefore, mobile phone is the ICT tool of choice for CHWs. Several studies provided evidence that mHealth tools can improve outcomes related to CHW performance such as quality of care, efficiency of services, CHW learning, and utilization of services. For example, a study used mobile multimedia devices to support point-of-care clinical decisions by CHWs in Colombia and found that CHWs had significantly fewer errors and better compliance with care protocols over a range of clinical care situations. The authors argued that although CHWs have little formal education and training, devices that use a combination of text, audio, images, and video can be used to improve their ability to provide quality community-based care [31]. However, it is important to note that if CHWs are to use mobile phones for nutrition service delivery, they would need to be supported with rigorous training as their current use of mobile phone is limited to sending and receiving voice calls. If smart phone-based application is called for, it is important that the cost of buying and using the mobile phone is taken into account in the design of the program as economic burden can be a serious barrier to the uptake and use of technology. Furthermore, it is important that technological platforms are seamlessly integrated into the workflow of the CHWs to avoid increasing workload and reducing acceptability.

In terms of health care seeking by the community members, researchers have reported that health care seeking in Bangladesh was quite actor centric [32] and mobile phones were largely used to connect with trusted source of health information and services. In our study we found that mothers were interested to be linked with the CHWs who were trusted community members. If we wanted to design a mobile phone-based system to reach the mothers and key family members with health information, it is important that the source of such information was perceived to be a trusted one. It is also important that the existing power dynamics in the households be taken into account when mobile phones are used to reach the mothers in the community. In the perspective of Bangladesh, involvement of husbands/partners in decision making is particularly important because in most families male figures play the dominant role in important household decision making such as income expenditure and healthcare-related movement [33]. Active involvement of these decision makers have been associated with increased utilization of maternal health services [34]. ICT tools such as mobile phones have been used very successfully in many low and middle income countries including Bangladesh, to remind mothers to use existing health services such as vaccination, antenatal care and other health services [26]. There is also some evidence that information provided to the community through mobile phones increased the knowledge and utilization of health services in low and middle income countries [35, 36]. However, existence of digital divide in terms of education, gender and socioeconomic status need to be taken into account if mobile phones are used to access mothers in rural communities in Bangladesh. Finally, it is important that opportunity for face-to-face counselling is not done away with if mobile phone is used by the frontline health workers as face-to-face counselling and active engagement with mothers have shown to improve nutrition related behaviours [8, 37, 38].

Despite the existing policy mandate for mainstreaming nutrition, our study showed that CHWs do not recognize their role in imparting nutrition services. In a recent review the researchers observed that community factors affecting CHW performance include cultural and gender norms, market forces and health system policy and practice. Our study showed that substantial work needs to be done to improve the implementation of nutrition services through CHWs by using some of the global best practices that improve CHWs motivation such as increasing their skills, providing incentives, and ensuring adequate monitoring and supervision [39]. The use of mobile phones can facilitate some of these processes. Globally the governments have started to act to achieve 17 Sustainable Development Goals (SDGs). Whilst the ambitious SDG 2 ‘End hunger, achieve food security and improved nutrition and promote sustainable agriculture’ is directly related to nutrition, at least 12 of the 17 Goals contain indicators that are highly relevant to nutrition [40]. Effective mainstreaming of nutrition services within the existing health systems is a key activity for achieving the SDGs. It is important therefore, that available technological platforms such as mobile phones be used when and where relevant to make the nutrition service delivery more efficient and effective. Meanwhile, it is also important that we consider community readiness to adopt technology during the design and implementation of the technological platforms.

The strength of our study is that it provided an understanding of the possibilities of using mobile phones for improving nutrition service delivery through CHWs and reaching out to the community in rural Bangladesh. However, this study, although rich in insight, was conducted in only two rural areas of Bangladesh and does not reflect the situation in all areas of Bangladesh.

## Conclusions

Despite high levels of mobile phone ownership reported in Bangladesh it was noted that mothers in rural areas have limited access and use of mobile phones. We found that the CHWs currently owned and used mobile phones both for personal and work purposes and were interested in using them to impart nutrition services to the community. Our study indicated that there is a potential to address some gaps in nutrition service delivery through ICTs such as mobile phones which are widely available. It is important however, to consider the readiness of the recipients to accept the technology during the design and delivery of the intervention.

## Abbreviations

ANC: Antenatal Care
CC: Community Clinics
CHCP: Community Health Care Providers
CHW: Community Health Workers
DGFP: Director General of Family Planning services
DGHS: Director General of Health Services
FGD: Focus Group Discussion
FP: Family Planning
FWA: Family Welfare Assistants
FWV: Family Welfare Visitors
HA: Health Assistant
HMIS: Health Management Information System
ICT: Information Communication Technology
IDI: In depth Interview
IYCF: Infant and Young Child Feeding
KII: Key Informant Interview
mHealth: Mobile Health
NGO: Non Government Organization
PNC: Postnatal Care
SDG: Sustainable Development Goal
SMS: Short Messages Service
UHFWC: Union Health Family Welfare Centre
